# ImmunoPET to help stratify patients for targeted therapies and to improve drug development

**DOI:** 10.1007/s00259-016-3458-6

**Published:** 2016-08-18

**Authors:** Françoise Kraeber-Bodere, Clément Bailly, Michel Chérel, Jean-François Chatal

**Affiliations:** 1 Inserm U892, CNRS UMR 6299, University Hospital-ICO-CRCNA, Nantes-Saint-Herblain, France; 2 Groupement d’Intérêt Public Arronax, University of Nantes, Nantes, France

Malignant tumours usually display intratumoral heterogeneity as well as phenotypic and genotypic heterogeneity among patients. Consequently, there is the need to develop treatments appropriate to each patient [1]. Screening of tumour phenotypes requires biopsy, a procedure that is invasive and limited to accessible tumour sites. Moreover, it is difficult to obtain repeated biopsies from the same lesions to explore changes in properties and heterogeneity during therapy. There is therefore the need for new noninvasive diagnostic technologies such as molecular imaging to assess whole-body tumour phenotypes to allow more specific therapeutic strategies to be developed.

There has been a considerable increase in the use of targeted therapies, including monoclonal antibodies (mAbs), in cancer management. A recent review found that there are more than 50 mAbs in advanced clinical development in oncology, including several antibody–drug conjugates and radiolabelled mAbs for radioimmunotherapy (RIT) [2]. Until now, only immunohistochemistry (IHC) analysis and quantitative polymerase chain reaction analysis of tumour biopsies have been able to identify patients with the highest chance of response to antibody-based therapy. However, these approaches do not allow whole-body mapping of tumour cell biomarker expression and do not assess biomarker accessibility.

mAbs can be labelled with radionuclides and are promising probes for theranostic approaches, offering a noninvasive solution to quantitatively assess in vivo target expression, to select patients for expensive and potentially toxic therapies and to monitor responses [3]. mAbs were initially labelled with single-photon emitters, such as ^131^I or ^111^In, and were subsequently used in planar imaging or SPECT imaging procedures to improve RIT using dosimetry procedures. Accurate quantitative information can be obtained more readily using PET. The good spatial resolution of PET allows better delineation of tumours and organs than with SPECT. Additionally, key factors for the superiority of PET over SPECT and planar imaging include exact attenuation correction, precise scatter correction and high sensitivity, combined with the possibility of performing true whole-body imaging in a reasonable time. Marrying mAbs and PET emitters requires an appropriate match between the biological half-life of the protein and the physical half-life of the isotope [4]. The use of ^18^F or ^68^Ga with a short half-life is limited to small molecules such as antibody fragments that distribute rapidly in the body, whereas ^89^Zr and ^124^I are well suited to the labelling of larger molecules such as intact immunoglobulins. ^64^Cu with an intermediate half-life of 12.7 h can be used for labelling a large number of molecules of different sizes.

In the present issue of *EJNMMI*, Sun et al. report the use of an anti-CD146 mAb labelled with ^64^Cu for quantitative immunoPET imaging of CD146 antigen expression in lung cancer models [5]. This antigen induces epithelial-to-mesenchymal transition, has a favourable receptor density expression (125,000 receptors per cell) and may be associated with the metastatic potential of cells and their resistance to apoptosis. Moreover, it has low expression levels in normal tissues. Therefore, a mAb specific for this antigen (YY146) has good potential for therapeutic application. In a preclinical study the authors assessed six human lung cancer cell lines with different expression levels of CD146 and showed a strong correlation between tumour uptake of ^64^Cu-NOTA-YY146 and relative expression of CD146 in the tumour cell lines. This radioimmunoconjugate is consequently appropriate for immunoPET for quantitative evaluation of CD146 expression in lung cancers before therapy using coupled or uncoupled YY146 antibody.

The first clinical proof that immunoPET is a powerful molecular diagnostic tool was reported by Divgi et al. The mAb girentuximab binds carbonic anhydrase IX, a cell-surface antigen highly and homogeneously expressed in more than 95 % of clear-cell renal cell carcinomas (ccRCC). In 26 presurgical patients with renal masses, immunoPET using ^124^I-girentuximab demonstrated a sensitivity of 94 % and a specificity of 100 %, with a negative predictive value of 90 % and a positive predictive value of 100 % [6]. These impressive results were corroborated in a phase III study, showing that ^124^I-girentuximab immunoPET discriminates the presence or absence of ccRCC with an accuracy at least comparable to that of biopsy analysis, suggesting that this invasive procedure with its inherent risks could be avoided [7].

Treatment strategies for individual patients could be tailored by using immunoPET. For example, anti-HER2 therapeutic agents are only effective in patients who have HER2-positive breast cancer as determined by IHC. It has been proven that mAbs labelled with ^68^Ga, ^64^Cu or ^89^Zr can noninvasively identify HER2-positive lesions and a few clinical studies have shown that immunoPET with ^89^Zr-mAbs is able to predict response to anti-HER2 antibody-based therapy [8–11]. In the ZEPHIR study, pretreatment PET using ^89^Zr-trastuzumab was assessed in 56 patients with IHC 3+ or FISH ≥2.2 HER2-positive metastatic breast cancer scheduled for treatment with trastuzumab emtansine (T-DM1) [12]. ^18^F-FDG PET was performed at baseline and before cycle 2 of T-DM1. The study showed 29 % negative HER2 PET/CT. Based on RECIST1.1. criteria, immunoPET showed a positive predictive value of 72 % and a negative predictive value of 88 %, and FDG PET a positive predictive value of 96 % and a negative predictive value of 83 %. The two imaging techniques combined gave a predictive value of 100 % and enabled patients with time to treatment failure of 2.8 months to be discriminated from those with time to treatment failure of 15 months.

In another study, the use of ^89^Zr-bevacizumab PET imaging for predicting response to combination therapy with carboplatin, paclitaxel and bevacizumab was assessed in seven patients with non-small-cell lung cancer. A positive but nonsignificant trend for a correlation between tumour uptake and progression-free and overall survival after treatment was found [13]. The same encouraging trend was found in ten patients with K-RAS advanced colorectal cancer who received ^89^Zr-cetuximab followed by treatment with cetuximab [14]. In other clinical applications such as ^89^Zr-bevacizumab followed by everolimus therapy in patients with neuroendocrine tumours [15], and ^89^Zr-fresolimumab followed by fresolimumab therapy in patients with high-grade glioma [16], no correlation was found between tumour uptake and clinical response. Based on these promising preliminary clinical results, it appears that immunoPET has a realistic potential for predicting responses to antibody-based therapy assuming that the biodistribution of the radioimmunoconjugate in immunoPET is the same as the biodistribution of the mAbs used for therapy. One serious drawback would be a negative immunoPET result predicting nonresponse to subsequent therapy in a patient who could have responded to the therapy, as has been shown in a few patients [14]. Randomized multicentre studies in stratified patients with different relevant indications are needed to demonstrate that immunoPET can be considered a true diagnostic companion.

Moreover, molecular in vivo imaging plays an increasing role in the development of new drugs by pharmaceutical companies. In vivo imaging is an effective solution for the rapid assessment of drug candidates, which may be radiolabelled to monitor their pharmacokinetics and biodistribution during preclinical and early clinical phases. Indeed, immunoPET is a powerful innovation to improve knowledge about the in vivo behaviour of mAbs, and provides information regarding the quantitative variation in molecular targets during treatments. ImmunoPET could provide information about tumour targeting, pharmacokinetics and accumulation in critical normal organs to determine optimal dosing and the impact of preloading with unlabelled antibody for RIT [17].

Consideration of the cost and safety of immunoPET is also important. A cost approaching several thousand euros per patient would be acceptable if the benefit in patient selection for expensive therapies and in drug development could be confirmed. Regarding dosimetry, the internal radiation doses estimated for immunoPET are comparable to those from conventional imaging and are acceptable. Due to a shorter physical half-life, the dose delivered with ^64^Cu is lower than that with ^89^Zr. Indeed, the internal radiation dose from ^64^Cu-trastuzumab absorbed by the patient has been estimated to be 4.5 mSv, compared with 18 mSv from ^89^Zr-trastuzumab [10]. Using activities ranging from 370 to 740 MBq, the radiation dose absorbed from ^18^F-FDG PET has been estimated to be 7 to 14 mSv.

In conclusion, we consider that immunoPET is a promising tool for personalized medicine, allowing better patient selection for antibody-based therapies and accelerating and improving drug development. Whilst this innovative technology is currently associated with a significant cost, this cost could become acceptable if the benefit in stratifying patients before expensive targeted therapies can be clearly demonstrated in large multicentre randomized clinical trials.
